# B cell-specific knockout of AID protects against atherosclerosis

**DOI:** 10.1038/s41598-023-35980-1

**Published:** 2023-05-30

**Authors:** Talin Ebrahimian, France Dierick, Vincent Ta, Maria Kotsiopriftis, Jonathan O’Connor Miranda, Koren K. Mann, Alexandre Orthwein, Stephanie Lehoux

**Affiliations:** Lady Davis Institute for Medical Research, 3755, Cote Ste Catherine, Montreal, QC H3T 1E2 Canada

**Keywords:** Cardiovascular diseases, B cells

## Abstract

Antigen-naive IgM-producing B cells are atheroprotective, whereas mature B cells producing class-switched antibodies promote atherosclerosis. Activation-induced cytidine deaminase (AID), which mediates class switch recombination (CSR), would thus be expected to foster atherosclerosis. Yet, AID also plays a major role in the establishment of B cell tolerance. We sought to define whether AID affects atherosclerotic plaque formation. We generated *Ldlr*^*-/-*^ chimeras transplanted with bone marrow from *Aicda*^*-/-*^ or wild-type (WT) mice, fed a HFD for 14 weeks. Decreased B cell maturation in *Ldlr*^*-/-*^*Aicda*^*-/-*^ mice was demonstrated by 50% reduction in splenic and aortic BAFFR expression, a key signaling component of B2 cell maturation. This was associated with increased plasma IgM in *Ldlr*^*–/-*^*Aicda*^*-/-*^ compared with *Ldlr*^*-/-*^WT animals. Importantly, *Ldlr*^*-/-*^*Aicda*^*-/-*^ mice had reduced atherosclerotic lesion area (0.20 ± 0.03mm^2^) compared with *Ldlr*^*-/-*^WT (0.30 ± 0.04mm^2^, P < 0.05), although no differences in plaque composition were noted between groups. In addition, immunofluorescence analysis revealed increased splenic B and T cell areas independent of cell number. AID depletion directly inhibits atherosclerotic plaque formation.

## Introduction

Atherosclerosis is an inflammatory disorder whereby fibrofatty plaques form and develop in the subendothelial space of arteries. Important encroachment of the vascular lumen by atherosclerotic lesions, or abrupt blockage of vessels due to plaque rupture, account for myocardial infarction and stroke. This is the predominant mechanism underlying cardiovascular disease, the leading cause of mortality globally^1^.

Lymphocytes play an important role in the formation and growth of atherosclerotic plaques. Although these lesions are characterized by a substantial accumulation of lipid laden macrophages and foamy cells of vascular smooth muscle origin, the inflammatory milieu promoted by B and T cells contributes significantly to disease progression. In particular, the involvement of B cells in atherosclerosis has recently received increased attention. B cells^2,3^ and antibodies targeting oxidation-specific epitopes (OSEs)^4^, such as oxidized low-density lipoprotein (oxLDL), have both been detected in human and murine atherosclerotic plaques. Caligiuri et al.^5^ showed that splenectomy aggravated atherosclerosis in *Apoe*^*-/-*^ mice on a high fat diet (HFD), which could be rescued by transfer of B cells from atherosclerotic mice that had been immunized against oxLDL. Likewise, Major et al.^6^ reported that bone marrow transplant (BMT) from B cell-deficient (*μMT*^*-/-*^) mice into HFD fed *Ldlr*^*-/-*^ recipients also increased atherosclerosis. While these initial findings suggested a generally protective role for B cells, subsequent experiments showed conflicting results. B cell depletion using anti-CD20 antibodies decreased plaques in experimental models of atherosclerosis^7,8^, and reconstitution with B2 cells, the conventional and most common B cell subtype, promoted atherosclerosis^8^. In comparison, B1a cells, which are predominantly depleted in splenectomized mice^9,10^, were found to be anti-atherogenic.

B1a and marginal zone B cells^11^ constitutively produce (i.e. independent of antigen stimulation^12,13^) most of the body’s antigen-naïve natural IgM antibodies^12^, many of which target OSEs and have been shown to protect against atherosclerosis^14–16^. In fact, IgM antibodies account for the atheroprotective effect of B1a cells^8,17^. In contrast, antibodies produced by follicular B2 cells are pro-atherogenic. B2 cell activation requires antigen recognition and T cell-dependent stimulation in secondary lymphoid organs such as the spleen and lymph nodes. Once activated, follicular B2 cells form germinal centers and undergo class switch recombination (CSR) to produce alternative immunoglobulin functional classes beyond the default IgM^18^. They also undergo somatic hypermutation (SHM) and affinity maturation, which refine target specificity through progressive rounds of variable region mutation and selection by follicular helper T cells for the B cell clones with the highest antigen affinity^19^.

Both CSR and SHM processes depend on the activation of a mutagenic enzyme called activation-induced cytidine deaminase (AID). Splenic B cells from *Aicda*^*-/-*^ mice that are stimulated to undergo CSR produce IgM and IgD but fail to produce antibody classes such as IgA and IgG. Conversely, overexpression of AID greatly increases CSR^20^. Furthermore, artificially expressing AID in non-B cell types allows them to undergo CSR and SHM^21–23^, demonstrating the potency of its effects. Loss-of-function *Aicda* mutations are associated with human immunodeficiency in the form of hyper-IgM syndrome^24^, whereas aberrant AID phenotypes (e.g. increased expression levels, targeting of non-immunoglobulin genes, expression in non-B cell types) are associated with diffuse large B cell lymphoma^25–27^, T cell lymphomas^28,29^, and other non-lymphoid tumors^30^. Given the major importance of AID in B2 cell maturation, and the pro-atherogenic effect of B2 cells, one would expect AID expression and activity to accelerate atherosclerosis. Yet AID also plays a major role in the establishment of both central and peripheral B-cell tolerance, promoting the removal of developing autoreactive B cells^31,32^, which would have the opposite effect on lesion formation.

The potential role of AID in atherosclerosis has not yet been extensively explored. We hypothesized that absence of AID, and associated modulation of B2 cell activation, impairs atherosclerosis. Here, we show that HFD upregulates splenic AID expression, and that transfer of *Aicda*^*-/-*^ lymphocytes in *Ldlr*^*-/-*^ mice reduces atherosclerotic plaque development.

## Results

### Validation of the experimental approach

We first investigated whether HFD regulates AID expression levels. *Ldlr*^*-/-*^ mice were fed a regular chow diet or fed a HFD for nine weeks (starting aged 8 weeks). AID protein expression was measured in spleen extracts. Compared to chow diet, the HFD had no impact on spleen AID expression, which was abundant in both groups (Suppl Fig. 1).

To verify proper reconstitution of the bone marrow following irradiation and to quantify the distribution and replacement of B and T cells, we performed bone marrow transplantation (BMT) using GFP + donor mice and WT recipients. Four weeks after lethal irradiation and BMT, virtually all circulating cells were GFP + , and over 90% of spleen cells were GFP + , as measured by flow cytometry (Suppl Fig. 2A). Furthermore, 98% splenic B cells and 75% splenic T cells were GFP + . White blood cell composition was also measured in the age matched donor and chimeric mice (Suppl Fig. 2B). Proportions of lymphocytes, monocytes, granulocytes and eosinophils were equivalent between groups. These results show efficient repopulation and support the notion that chimeric mice receiving *Aicda*^*-/*-^ bone marrow have AID-depleted B cells.

### Depletion of AID attenuates atherosclerotic plaque development

**Figure 1:**
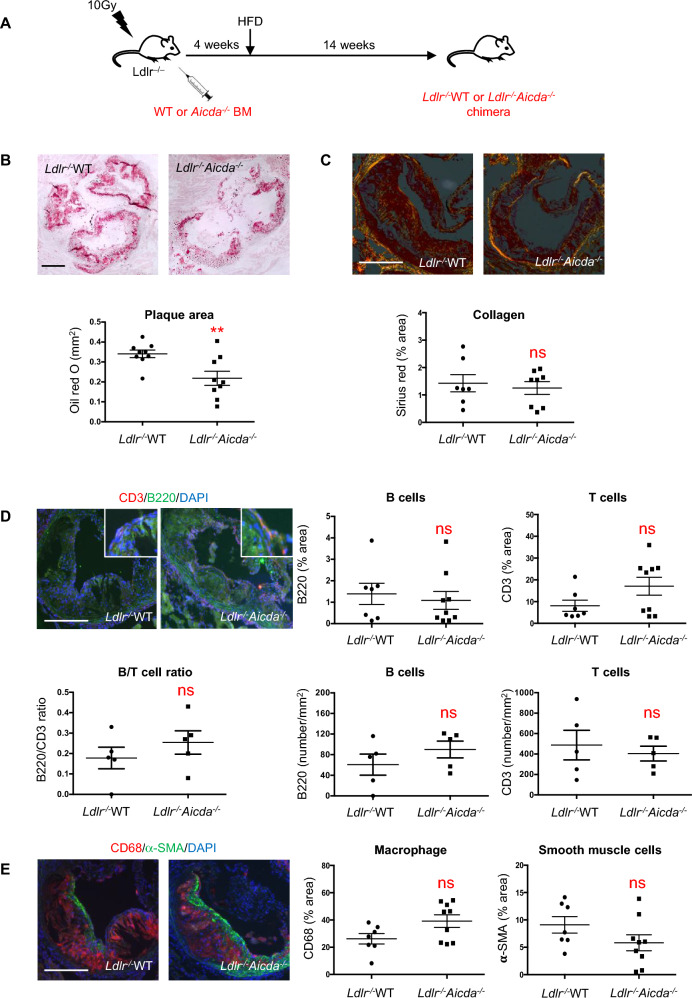
Bone marrow cell depletion of AID reduces plaque progression but does not affect plaque composition. (**A**) Outline of bone marrow transfer (BMT) experiment: *Ldlr-/-* mice were subjected to 10 Gy of total body irradiation. The following day, they received retro-orbital injections of bone marrow from either wild-type (WT) or *Aicda*^*–/–*^ donors to form chimeric mice. After 4 weeks of recovery, they were given HFD for 14 weeks. (**B**-**E**) Representative histological and immunofluorescent images and quantifications of aortic sinus plaque composition from *Ldlr*^*-/-*^WT and *Ldlr*^*-/-*^*Aicda*^*-/-*^ chimeric mice: (B) plaque size assessed by Oil Red O staining; (**C**) collagen I/III evaluated by Picrosirius Red staining; immunofluorescent probing for (**D**) CD3^+^ T lymphocytes (red), B220^+^ B cells (green), (**E**) CD68^+^ macrophages (red), and α-smooth muscle actin (α-SMA)^+^ smooth muscle cells (green). Results are expressed as number of cells/mm^2^ plaque area for B220 + /CD3 + cells and as % of plaque area for CD68/a-SMA. Data are mean ± SEM of n = 7–9. ** P < 0.01 vs *Ldlr*^*-/-*^WT. Scale bar = 50 μM.

To investigate whether AID regulates atherosclerosis, we transplanted bone marrow cells from *Aicda*^*-/-*^ mice or wild-type (WT) littermates into irradiated *Ldlr*^*-/-*^ mice and then fed both groups a HFD for 14 weeks (Fig. 1A). Compared with *Ldlr*^*-/-*^WT, aortic sinus lesion areas in *Ldlr*^*-/-*^*Aicda*^*-/-*^ chimeric mice were reduced by one third (P < 0.01) (Fig. 1B). Despite this dramatic difference in plaque size, no other dissimilarities in plaque composition were noted between *Ldlr*^*-/-*^WT and *Ldlr*^*-/-*^*Aicda*^*-/-*^ chimeric mice. Relative lesion collagen content was equivalent between the two groups (Fig. 1C), as was cellular composition as regards B220 + , CD3 + lymphocytes (Fig. 1D), CD68 + foam cells, and smooth muscle cells (Fig. 1E). In addition, flow cytometry revealed that in both chimeric groups, 2–5% of aortic cells were B220^+^ B cells (data not shown). These results indicate that absence of AID impairs plaque development but does not influence immune cell composition or lesion stability.

In both the spleen and the aorta, we observed a significant ~50% reduction in BAFF receptor expression in *Aicda*^*-/-*^*Ldlr*^*-/-*^ chimeras compared with *Ldlr*^*-/-*^WT chimeras (Suppl Fig. 3A). This duplicated observations in age-matched *Aicda*^*-/-*^ vs WT littermates (not shown), confirming the defect in the B cell activation process in the absence of AID. Interestingly, loss of BAFFR signaling^33,34^ or BAFFR deficiency^35^ were previously reported to reduce atherosclerosis progression. No differences in total cholesterol, LDL, HDL, or triglyceride concentrations were observed between the groups (Suppl Fig. 3B). Similarly, animals displayed equivalent weight gain (Suppl Fig. 3C).

### Depletion of AID increases IgM production and regulates splenic B cell populations

**Figure 2:**
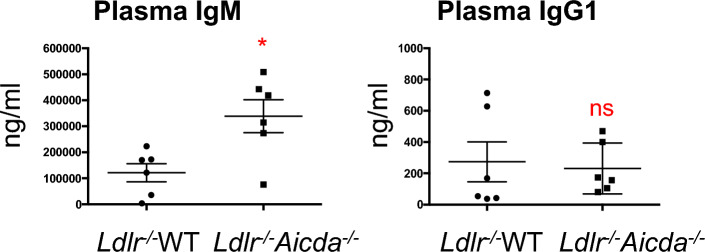
Bone marrow cell depletion of AID increases plasma IgM levels without impacting plasma IgG levels. Plasma IgM and IgG1 quantifications. Results are expressed as ng/ml. Data are mean ± SEM of n = 6. *P < 0.05 vs *Ldlr*^*-/-*^WT.

As could be anticipated, *Ldlr*^*-/-*^*Aicda*^*-/-*^ chimeric mice displayed increased plasma IgM levels compared with *Ldlr*^*-/-*^WT (339 ± 63 vs 121 ± 35 ng/ml (× 10^3^), respectively, P < 0.05) (Fig. 2). This paralleled the elevated IgM levels observed in the *Aicda*^*-/-*^ donor mice compared with WT (443 ± 121 vs 86 ± 48 ng/ml (× 10^3^), respectively, P < 0.01) (Suppl Fig. 4A). Nevertheless, despite dramatically lower plasma IgG1 levels in *Aicda*^*-/-*^ donor mice compared to WT littermates (0.3 ± 0.03 vs 3.7 ± 1.4 ng/ml (× 10^3^), respectively, P < 0.01) (Suppl Fig. 4A), this characteristic was not replicated in the chimeras (0.27 ± 0.1 vs 0.23 ± 0.7 ng/ml (× 10^3^)) (Fig. 2).

**Figure 3:**
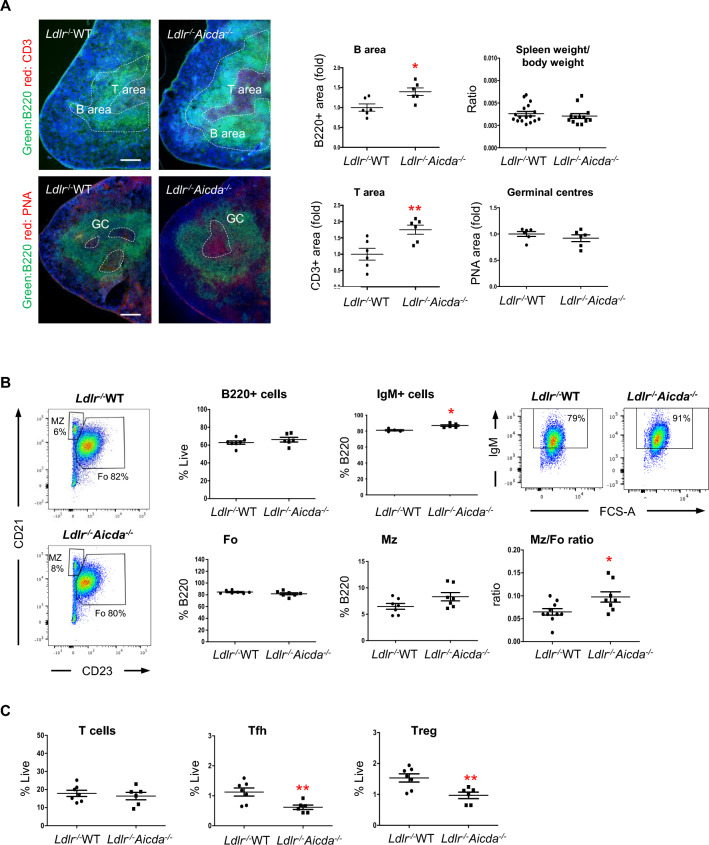
Bone marrow cell depletion of AID expands spleen B and T cell areas and reduced mature T cell populations. **(A)** Representative immunofluorescent images and quantifications of spleen reveal expanded B220 + B cell (green) and CD3 + T cell (red) areas in *Ldlr*^*-/-*^*Aicda*^*-/-*^ mice compared with *Ldlr*^*-/*-^WT. However, spleen weight measured as a ratio of total body weight does not differ between chimeric groups. Also, no differences in spleen germinal center area (PNA-red) within B cell area (B220-green) are found between groups. Nuclei are stained with Dapi (blue). **(B)** Flow cytometry quantifications of total B220 + and IgM + B cells (among B220 +), as well as follicular (Fo: B220 + CD21-CD23 +) and marginal zone (Mz: B220 + CD21intCD23-) B cells and Mz/Fo ratio in the spleen. **(C)** Flow cytometry of total CD4 + T cells as well as T follicular helper (Tfh: CD4 + PD1 + CXCR5 +) and regulatory T cells (Treg: CD4 + CD25 + Foxp3 +) T cells. Immunofluorescence quantifications are expressed as fold difference with respect to mean of the *Ldlr*^*-/-*^WT chimeras. Flow cytometry results are expressed as a proportion of live cells except for IgM + B cells. Data are mean ± SEM of n = 6–7. *P < 0.05 and **P < 0.01 vs. *Ldlr*^*-/-*^WT. Scale bar = 50 μM.

Since AID acts mainly in secondary lymphoid organs during adaptive immune responses, we assessed splenic immune cell subtypes by immunocytochemistry and flow cytometry. There was no difference in spleen weight/body weight ratios between both groups of chimeric mice (Fig. 3A, Suppl Fig. 4B). However, spleens from the *Ldlr-/- Aicda*^*-/-*^ chimeric mice displayed a marked follicle expansion, with increased T- and B-cell zones (Fig. 3A), as reported earlier in *Aicda*^*-/-*^ mice^36^. Nevertheless, germinal center areas within B220 + B cell area, determined by peanut agglutinin (PNA) staining, were equivalent between both groups (Fig. 3A). Also, flow cytometry of the spleen revealed no overt differences in the proportions of B220^+^ B or CD4^+^ T cells between *Ldlr*^*-/-*^WT and *Ldlr*^*-/-*^*Aicda*^*-/-*^ chimeric mice (Fig. 3B-C), mimicking data obtained in the donor WT and *Aicda*^*-/-*^ mice (Suppl Fig. 3C). Likewise, relative proportions of follicular and marginal zone B cells were equivalent in both chimeric groups (Fig. 3B), and proportions of naïve B1 cells were equivalent between both groups (not shown). However, IgM^+^ B220^+^ B cells and the Mz/Fo B cell ratio were significantly increased in *Ldlr*^*-/-*^*Aicda*^*-/-*^ chimeric vs *Ldlr-/-*WT mice, indicating an altered splenic B cell composition toward IgM-expressing B cells. Also, despite an equal total CD4^+^ T cell numbers in the two chimeric groups, different subtypes of T cells such as regulatory T cells (Tregs) and T follicular helper cells (Tfh) were significantly decreased in *Aicda*^*-/-*^ compared to WT chimeras (Fig. 3C). Our results suggest that compared to WT littermates, *Aicda*^*-/-*^ chimeras exhibit a modification of spleen B and T cell area architecture in the context of inflammation, as well as modulated T cell differentiation.

## Discussion

Experimental animal and clinical studies reported conflicting data regarding the contribution of B cells to atherosclerosis and cardiovascular diseases^37^. B cell responses associated with atherosclerosis have mainly implicated the antibody response to oxLDL. In the presence of a specific antigen, AID enzyme is activated in B cells and allows them to undergo somatic hypermutation and CSR, two important processes increasing the affinity of the antibodies for a given antigen, essential for the adaptive immune response. We found that atherosclerotic lesions were significantly smaller in the aortic sinus of *Ldlr*^*-/-*^ mice transplanted with the bone marrow from *Aicda*^*-/-*^ mice compared with WT mice. Despite the important reduction in plaque development, no other differences in lesion composition, either cellular or structural, were noted between the chimeric groups.

Reduced plaque size in *Ldlr*^*-/-*^*Aicda*^*-/-*^ chimeric mice was accompanied by an increase in plasma total antigen naïve IgM with no difference in IgG1 levels. Importantly, IgM levels in age matched *Aicda*^*-/-*^ mice were also elevated as compared to controls, indicating that IgM levels are regulated by AID independently from HFD stimulation. However, since IgMs are known to be atheroprotective antibodies^17^, their increase in *Aicda*^*-/-*^ chimeric mice may be responsible for reducing plaque development by neutralizing oxidized LDL particles. It is important to note that total plasma IgM was measured here, but a recent publication confirmed that *ApoE*^*-/-*^*Aicda*^*-/-*^ mice developed IgM antibodies targeting malondialdehyde-oxidized low density lipoprotein and -ApoB100^25^. Previously, Kyaw et al.^16^ demonstrated that the atheroprotective properties of B1a cells are mediated by production of IgM. Whereas transfer of WT B1a cells into splenectomized *ApoE*^-/-^ mice reduced splenectomy-driven lesion progression, transfer of soluble IgM^-/-^ (secretion-null) B1a cells failed to do so. Splenectomy also reduced plaque IgM deposition, which was restored in mice injected with WT B1a cells. Furthermore, splenic marginal zone B cells have been shown to produce IgM fighting against blood borne pathogens. All these data are in favour of an atheroprotective role of IgM antibodies. Plasma IgM levels were doubled in *Aicda*^*-/-*^ chimeric compared to WT mice This could be explained by amplified splenic Mz/Fo B cell ratio, as well as augmented IgM-expressing B220 + cells in the spleen. Increased IgM levels could also be explained by disrupted CSR in *Aicda*^*-/-*^ chimeric mice. However, impaired CSR would also be expected to decrease plasma IgG1, but this was not observed in the *Aicda*^*-/-*^ chimeric mice, though there was a decrease in plasma IgG1 in the age-matched native *Aicda*^*-/-*^ mice. While the role of the IgG isotype antibodies in atherosclerosis remains controversial^37^, the fact that they have a broad range of functions and constitute the majority of circulating antibodies warrants further investigation into the relationship between AID and IgG.

In spleen follicles, the T cell-dependent adaptive immune response to specific antigens leads to antibody production^38–42^. Within the germinal center, IgM^+^ B cells capture the T-dependent antigen on their B cell receptor, migrate to and interact with antigen specific Tfh cells for affinity selection. In the *Ldlr*^*-/-*^*Aicda*^*-/-*^ chimerics, we did not observe a decreased in splenic GC formation. Interestingly, immunofluorescent staining showed an increase in splenic B and T cell areas in these mice. This suggests a modification of spleen architecture. Given that in the absence of AID there is low or no CSR^27^, an essential component of T cell-dependent B cell activation, this could lead to splenic accumulation of B cells (and their partner T cells) that are unable to complete their activation process. Indeed, our flow cytometry experiments on spleen showed equivalent proportions of B and T cells in both chimeric groups but reduced Tfh and Treg subtypes in *Ldlr*^*-/-*^*Aicda*^*-/-*^ mice compared with *Ldlr*^*-/-*^WT mice. Since these T cell subtypes have opposing effects on atherosclerosis, it is difficult to conclude on their participation to lesion formation in our mice.

Absence of B cell activation/maturation in native *Aicda*^*-/-*^ as well as chimeric *Aicda*^*-/-*^ mice was associated with a marked reduction of BAFF-R expression in the spleen and the aorta. B cells have been detected in vessel wall adventitia^43^, and they were also found in early fatty streak–type lesions as well as in well-established atherosclerotic plaques^2^. Interestingly, these artery tertiary lymphoid organ B cells were predicted to orchestrate atherosclerotic B cell immunity^44^. BAFF-R is one of three known receptors for BAFF ligand expressed mainly on mature B cells and is a critical regulator of B- and T-cell function^45^. Inhibiting BAFF-R with a monoclonal antibody^33,34^ or genetic knockout^35^ impairs B2 cell maturation, reducing atherosclerosis. Indeed, BAFF-BAFFR interaction is required for isotype-switching and enhances antibody production by B cells. Although our data do not demonstrate a direct role for AID in regulating BAFF-R, reduced expression of this receptor in *Ldlr*^*-/-*^*Aicda*^*-/-*^ chimeric mice provides evidence of reduced B cell activation or maturation, which in turn could help explain the reduced plaque size in these mice.

Our results, along with previous findings, suggest that absence of AID in bone marrow derived-B cells reduces development of atherosclerotic lesions possibly by preventing CSR and/or SHM, leading to impairment of B cell maturation/activation and partly by shifting the humoral balance towards increased atheroprotective natural IgM. Our work agrees with reports that B cell depletion therapy is effective against atherosclerotic plaque development^46^. A unique aspect of atherosclerosis is the prominent role of natural antibodies, specifically those binding to the oxidized epitopes that are abundant on modified lipoproteins and cellular debris. Our study reinforces the precept that this is a promising therapeutic avenue to develop.

## Methods

According to the guidelines of the McGill University animal committee and the Canadian Council on Animal Care, the mice are first anesthetised with isoflurane and then euthanized by CO2. We confirm that all methods are reported in accordance with ARRIVE guidelines (https://arriveguidelines.org) for the reporting of animal experiments.

### Mice and treatments

C57BL/6 and *Ldlr*^*-/-*^ were obtained from Jackson Laboratory (Bar Harbor, ME). *Aicda*^*-/-*^ mice were a generous gift from Dr. Di Noia (Montreal Clinical Research Institute). All animal experiment protocols were approved by the McGill University Animal Care Committee and animals were handled in accordance with institutional guidelines. Both strains were bred in house and verified for the correct genotype by PCR analysis of ear tag DNA extracts.

Eight-week-old *Ldlr*^*-/-*^ mice were placed on either normal (chow) or high fat diet for nine weeks and then euthanized for sample collection. Upon sacrifice, blood was obtained by cardiac puncture and collected in EDTA-coated tubes (Sarstedt, Nümbrecht, Germany). Spleen and aorta samples were lysed, and protein collected to quantify expression of AID by Western blot.

### Bone marrow transplantation

To selectively knock out *AID* in B cells in a proatherogenic mouse model, we performed bone marrow transfer (BMT) experiments (Fig. 1A). Six-week-old *Ldlr*^*-/-*^ mice were immunosuppressed by lethal total body irradiation (10 Gy-Andrex Smart 225 Röntgen source, YXLON International, Hamburg, Germany)^41^. The following day, they received a retro-orbital injection of total 10^6^ bone marrow cells harvested from either *Aicda*^*-/-*^ mice or wild-type littermates (age-matched) to reconstitute depleted bone marrow cells. After 4 weeks of recovery, they were started on a high fat diet (HFD) for 14 weeks and were then euthanized for sample collection. Some of the experiments described below were also performed in parallel in age-matched (26-week-old) non-BMT *Aicda*^*-/-*^ and wild-type mice.

To quantify the proportion of B and T cells in recipient mice upon bone-marrow transplantation, similar BMT experiments were performed using GFP + bone marrow donors and WT recipients (Suppl Fig. 2A). Mice were sacrificed 4 weeks after irradiation and transplantation, and flow cytometry was performed on spleen samples.

The repopulation efficacy was verified by counting the proportion of total white blood cells in age matched donor and recipient mice upon BMT (Suppl Fig. 2B) using Scil Vet ABC (Laboratory Diagnostics).

### Plasma lipids and IgM/IgG analysis

Plasma lipids were measured by McGill diagnostic laboratory. Plasma IgM and IgG1 levels were measured in the plasma of mice using an Elisa kit (eBiosciences, NY) according to the manufacturer’s protocol.

### Immunohistochemistry

Spleen and heart samples were OCT-embedded for sectioning (6 μm). The aortic sinus sections were stained with Oil Red O (Electronic Microscopy Sciences, PA) using brightfield microscopy and analyzed for lesion area, or were stained with Picosirius Red (Polysciences, PA) for collagen content, measured under polarized light. CD68 (Biolegend, CA), α-SMA (Sigma, ON), CD3 (Agilent, CA), or B220 (Biolegend) immunostainings were used in the aortic sinus or spleen sections to determine macrophage, smooth muscle cell, T cell, and B cell contents, respectively. Spleen sections were incubated with rhodamine coupled PNA (Vector Laboratories, Burlingame, USA) to detect germinal centers.

### Western blotting

RIPA-soluble proteins (20 μg) from spleen or total aorta were separated by SDS-PAGE, transferred to nitrocellulose membranes, and probed with anti-AID, anti-BAFFR (Abcam, MA) and anti-β-actin antibodies (Santa Cruz Biotechnology, TX) overnight at 4 °C. Regarding the western blot images in Supplemental Fig. 1, the membrane was cut at 35 kDa, allowing for incubation of the upper part with anti-beta-actin and the lower part with anti-AID antibodies.

Membranes were washed three times with TBST and incubated with HRP-conjugated secondary antibody (Bio-Rad Laboratories, CA). Membranes were revealed by chemiluminescence with the ChemiDoc™ MP Imaging System (Bio-Rad Laboratories, CA) and quantified by densitometry using Quantity One software (Bio-Rad).

### Immune cell phenotyping

Splenocytes were prepared by gently mincing the spleen and filtering the suspension through a cell strainer (100 μm pores, BD Biosciences). Aortas were isolated and digested for one hour at 37 °C in Liberase™ protease solution (Hoffmann-La Roche Ltd., Basel, Switzerland). Cells were centrifuged for 5 min at 400 g, re-suspended in 4% FcR blocking reagent (MACS Miltenyi Biotec, Bergisch Gladbach, Germany), and stained with antibodies targeting the surface markers B220, IgM, CD4, TCR-β. Flow cytometry was performed on the BD LSR Fortessa (BD Biosciences, CA). Fluorescence minus one (FMO) controls were used to determine fluorescence background and positivity. Data analysis was performed using Flow Jo software (BD Biosciences). Gating was first performed on forward versus side scatter to remove cell debris and doublets before selection of live cells based on exclusion of a viability dye, LIVE/DEAD™ Fixable Aqua (Invitrogen, CA). Gating strategies for all flow cytometry data are presented in Suppl Fig. 5.

### Statistical analysis

Data were analysed in Prism (GraphPad Software Inc., CA). Data are presented as mean ± SEM or fold difference relative to the mean of the control condition for each experiment day, and the Mann–Whitney U test was used to compare means. Outliers were identified using the ROUT method.

## Supplementary Information

Supplementary Information.

## Data Availability

All data generated or analysed during this study are included in this published article [and its supplementary information files].
