# Supplementary material for: B cell-specific knockout of AID protects against atherosclerosis
Source: Sci Rep. 2023 May 30;13:8723. doi: 10.1038/s41598-023-35980-1 (PMC10229602; doi:10.1038/s41598-023-35980-1)
Supplement: Supplementary file 1 — Supplementary Information. [file 41598_2023_35980_MOESM1_ESM.pptx]

## Slide 1
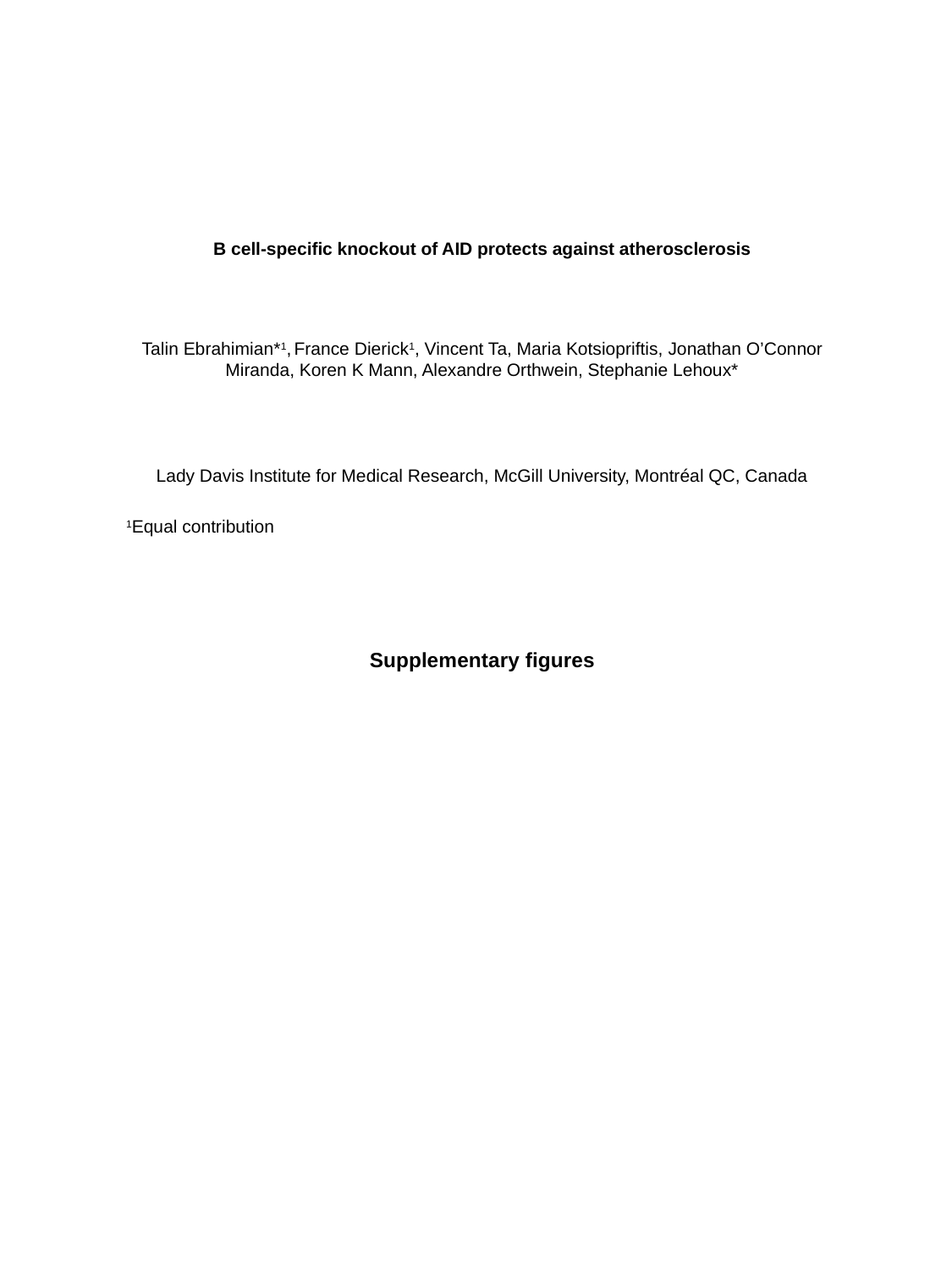

B cell-specific knockout of AID protects against atherosclerosis
Talin Ebrahimian*1, France Dierick1, Vincent Ta, Maria Kotsiopriftis, Jonathan O’Connor Miranda, Koren K Mann, Alexandre Orthwein, Stephanie Lehoux*
Lady Davis Institute for Medical Research, McGill University, Montréal QC, Canada
1Equal contribution
Supplementary figures

## Slide 2
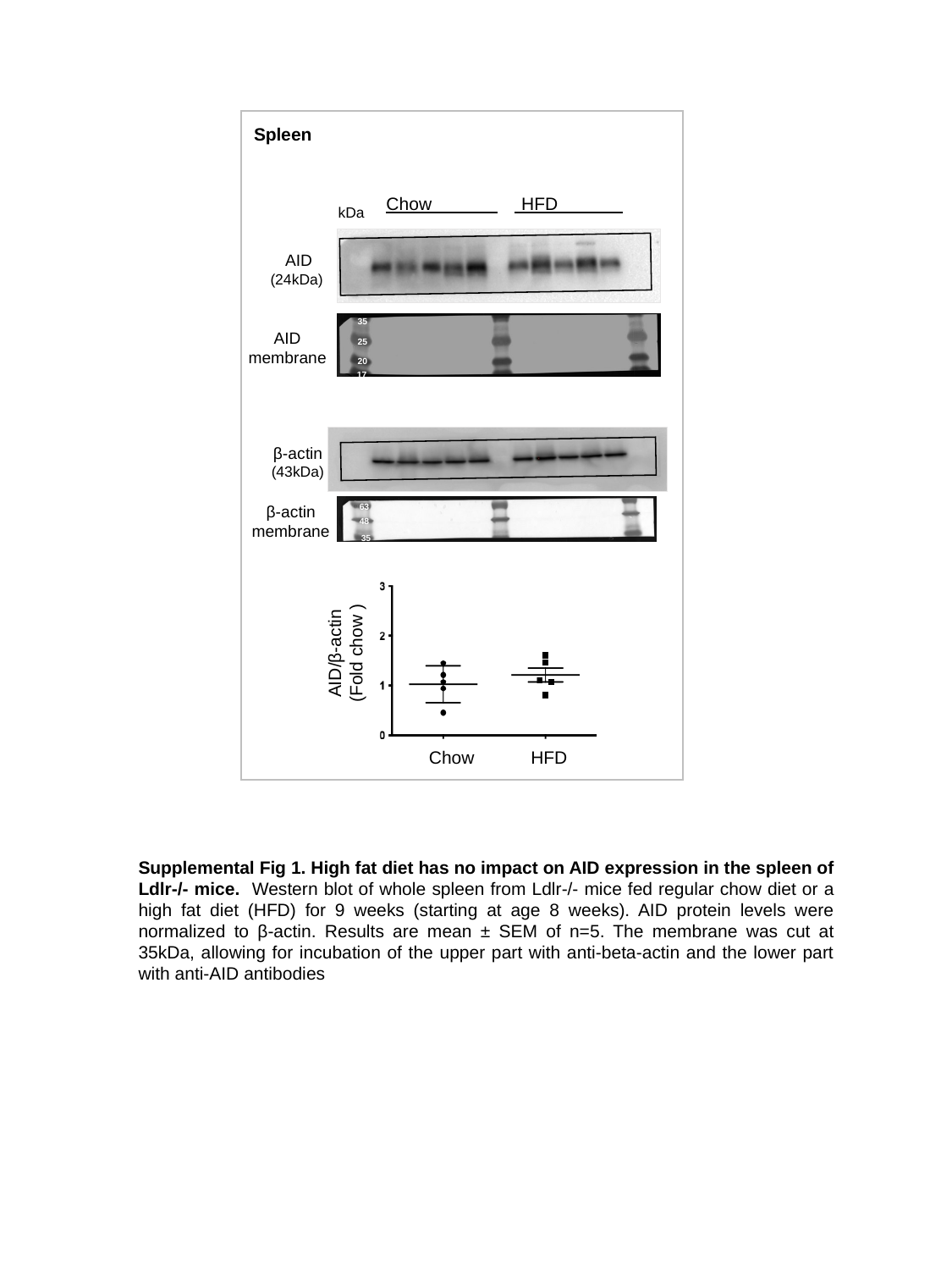

Spleen
Chow
HFD
35
35
35
25
25
25
20
20
20
17
17
17
10
63
63
48
48
35
35
kDa
AID
(24kDa)
AID
membrane
β-actin
(43kDa)
β-actin
membrane
 AID/β-actin
(Fold chow )
Chow
HFD
Supplemental Fig 1. High fat diet has no impact on AID expression in the spleen of Ldlr-/- mice. Western blot of whole spleen from Ldlr-/- mice fed regular chow diet or a high fat diet (HFD) for 9 weeks (starting at age 8 weeks). AID protein levels were normalized to β-actin. Results are mean ± SEM of n=5. The membrane was cut at 35kDa, allowing for incubation of the upper part with anti-beta-actin and the lower part with anti-AID antibodies

## Slide 3
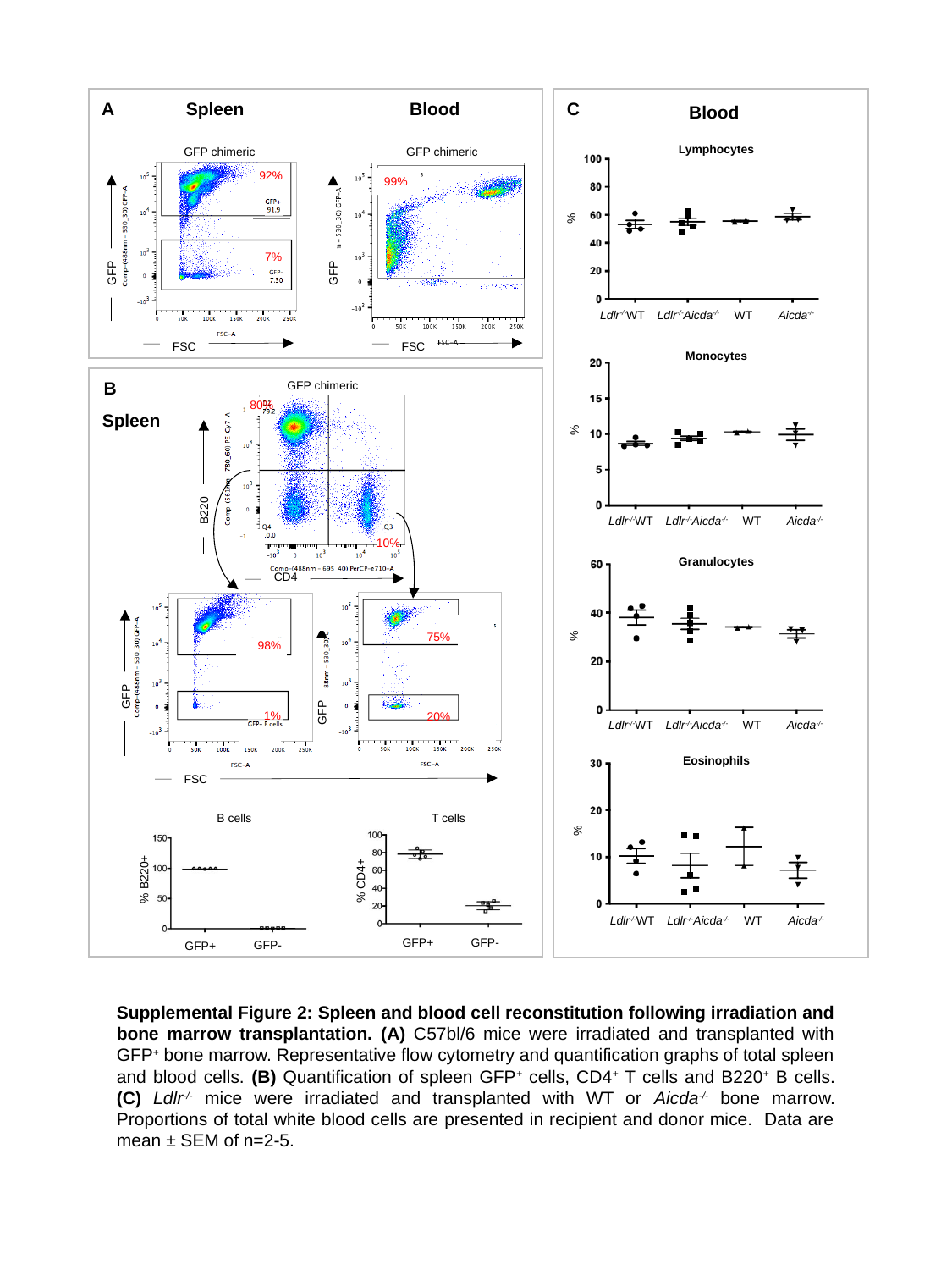

Spleen
Blood
 GFP chimeric
 GFP chimeric
92%
7%
99%
%
GFP
GFP
FSC
FSC
A
C
Blood
 Lymphocytes
Ldlr-/-WT
Ldlr-/-Aicda-/-
WT
Aicda-/-
Monocytes
%
Ldlr-/-WT
Ldlr-/-Aicda-/-
WT
Aicda-/-
Granulocytes
Ldlr-/-WT
Ldlr-/-Aicda-/-
WT
Aicda-/-
Eosinophils
%
Ldlr-/-WT
Ldlr-/-Aicda-/-
WT
Aicda-/-
B
 GFP chimeric
80%
B220
10%
CD4
75%
98%
GFP
1%
20%
FSC
B cells
T cells
% B220+
% CD4+
GFP+
GFP-
GFP-
GFP+
GFP
Spleen
%
Supplemental Figure 2: Spleen and blood cell reconstitution following irradiation and bone marrow transplantation. (A) C57bl/6 mice were irradiated and transplanted with GFP+ bone marrow. Representative flow cytometry and quantification graphs of total spleen and blood cells. (B) Quantification of spleen GFP+ cells, CD4+ T cells and B220+ B cells. (C) Ldlr-/- mice were irradiated and transplanted with WT or Aicda-/- bone marrow. Proportions of total white blood cells are presented in recipient and donor mice. Data are mean ± SEM of n=2-5.

## Slide 4
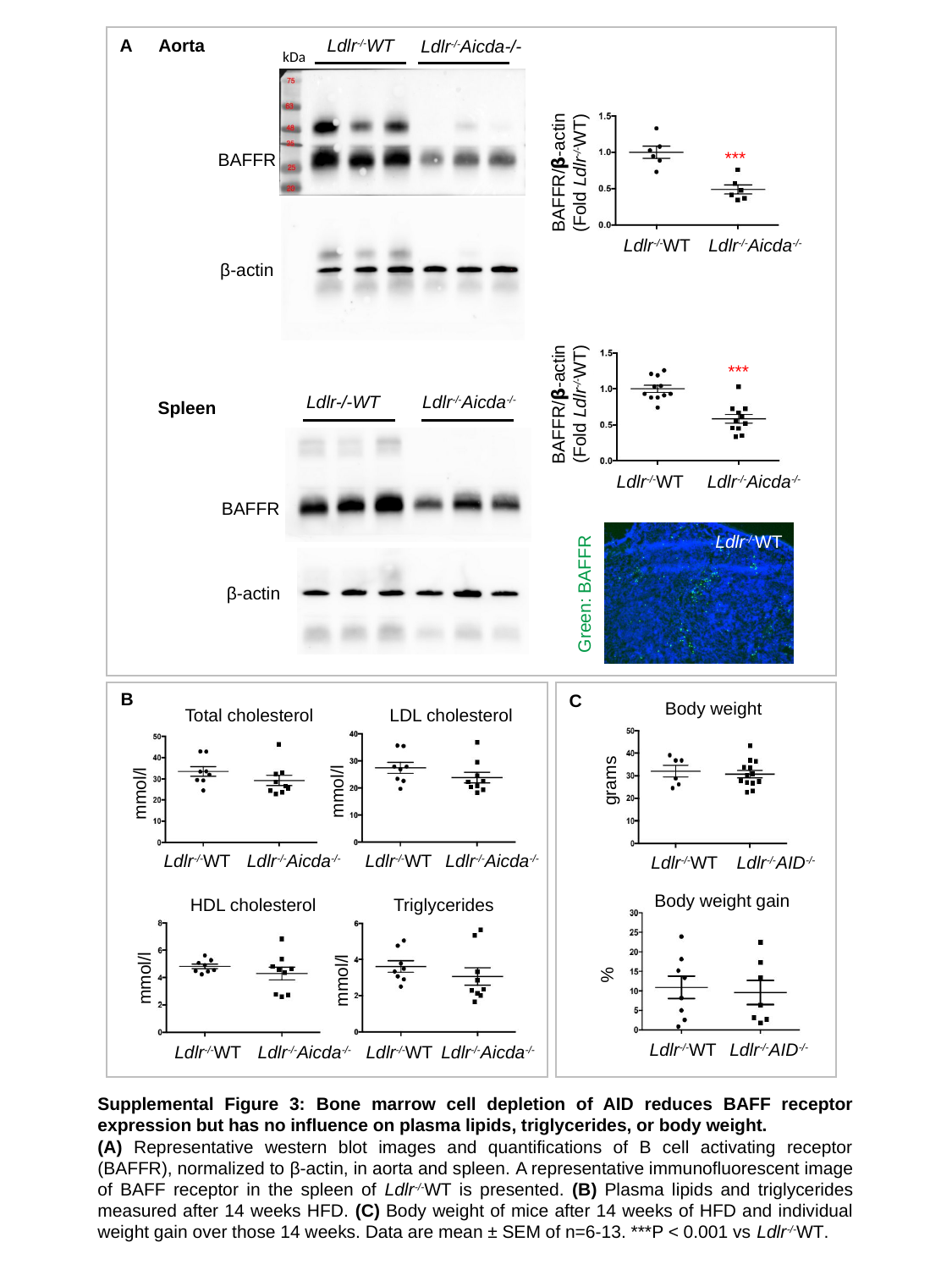

A
Aorta
Ldlr-/-WT
Ldlr-/-Aicda-/-
kDa
BAFFR/𝝱-actin
(Fold Ldlr-/-WT)
***
BAFFR
Ldlr-/-WT
Ldlr-/-Aicda-/-
β-actin
***
BAFFR/𝝱-actin
(Fold Ldlr-/-WT)
Ldlr-/-WT
Ldlr-/-Aicda-/-
Spleen
Ldlr-/-WT
Ldlr-/-Aicda-/-
BAFFR
Ldlr-/-WT
Green: BAFFR
β-actin
B
C
Body weight
Total cholesterol
LDL cholesterol
grams
mmol/l
mmol/l
Ldlr-/-WT
Ldlr-/-Aicda-/-
Ldlr-/-WT
Ldlr-/-Aicda-/-
Ldlr-/-WT
Ldlr-/-AID-/-
Body weight gain
HDL cholesterol
Triglycerides
%
mmol/l
mmol/l
Ldlr-/-WT
Ldlr-/-AID-/-
Ldlr-/-WT
Ldlr-/-Aicda-/-
Ldlr-/-WT
Ldlr-/-Aicda-/-
Supplemental Figure 3: Bone marrow cell depletion of AID reduces BAFF receptor expression but has no influence on plasma lipids, triglycerides, or body weight.
(A) Representative western blot images and quantifications of B cell activating receptor (BAFFR), normalized to β-actin, in aorta and spleen. A representative immunofluorescent image of BAFF receptor in the spleen of Ldlr-/-WT is presented. (B) Plasma lipids and triglycerides measured after 14 weeks HFD. (C) Body weight of mice after 14 weeks of HFD and individual weight gain over those 14 weeks. Data are mean ± SEM of n=6-13. ***P < 0.001 vs Ldlr-/-WT.

## Slide 5
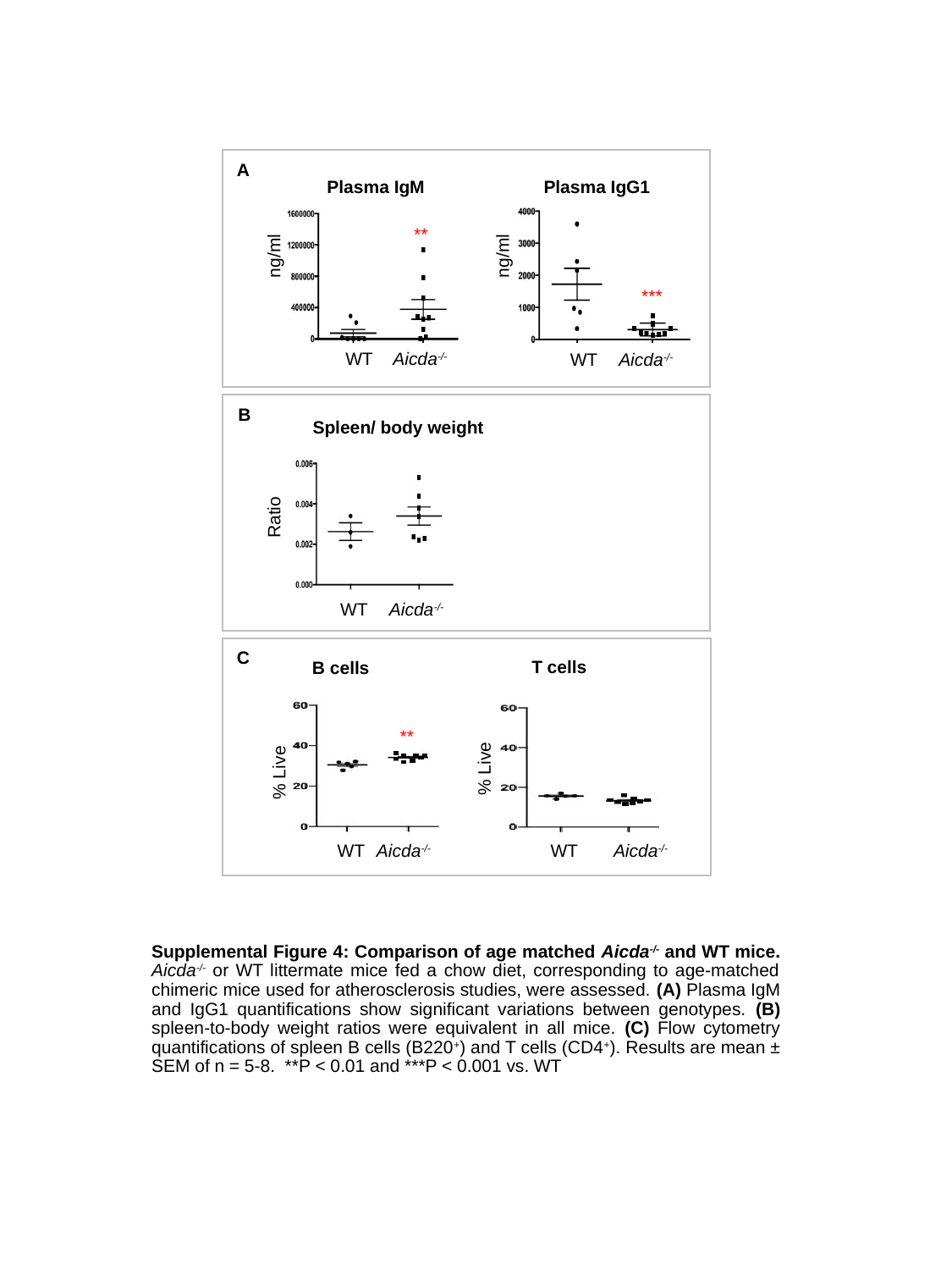

A
Plasma IgM
Plasma IgG1
**
ng/ml
ng/ml
***
WT
Aicda-/-
WT
Aicda-/-
B
Spleen/ body weight
Ratio
WT
Aicda-/-
C
T cells
B cells
**
% Live
% Live
WT
Aicda-/-
WT
Aicda-/-
Supplemental Figure 4: Comparison of age matched Aicda-/- and WT mice. Aicda-/- or WT littermate mice fed a chow diet, corresponding to age-matched chimeric mice used for atherosclerosis studies, were assessed. (A) Plasma IgM and IgG1 quantifications show significant variations between genotypes. (B) spleen-to-body weight ratios were equivalent in all mice. (C) Flow cytometry quantifications of spleen B cells (B220+) and T cells (CD4+). Results are mean ± SEM of n = 5-8. **P < 0.01 and ***P < 0.001 vs. WT

## Slide 6
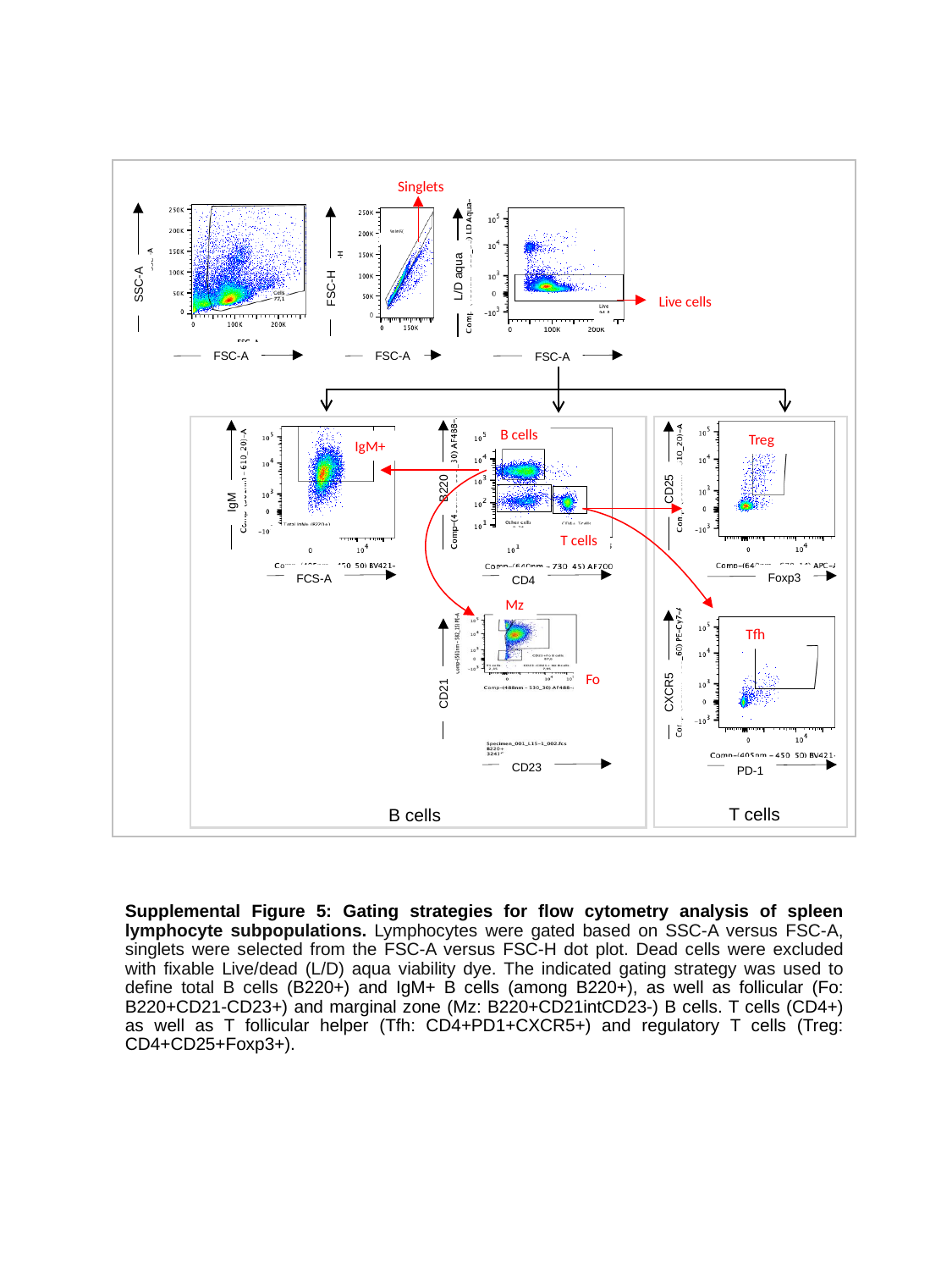

Singlets
L/D aqua
SSC-A
FSC-H
Live cells
FSC-A
FSC-A
FSC-A
B cells
Treg
IgM+
B220
CD25
IgM
T cells
v
v
Foxp3
FCS-A
CD4
Mz
Tfh
Fo
CXCR5
CD21
CD23
PD-1
T cells
B cells
Supplemental Figure 5: Gating strategies for flow cytometry analysis of spleen lymphocyte subpopulations. Lymphocytes were gated based on SSC-A versus FSC-A, singlets were selected from the FSC-A versus FSC-H dot plot. Dead cells were excluded with fixable Live/dead (L/D) aqua viability dye. The indicated gating strategy was used to define total B cells (B220+) and IgM+ B cells (among B220+), as well as follicular (Fo: B220+CD21-CD23+) and marginal zone (Mz: B220+CD21intCD23-) B cells. T cells (CD4+) as well as T follicular helper (Tfh: CD4+PD1+CXCR5+) and regulatory T cells (Treg: CD4+CD25+Foxp3+).
